# Influence of mouth breathing on outcome of scaling and root planing in chronic periodontitis

**DOI:** 10.1038/s41405-018-0007-3

**Published:** 2018-11-09

**Authors:** Manpreet Kaur, Rajinder Kumar Sharma, Shikha Tewari, Satish Chander Narula

**Affiliations:** Department of Periodontics and Oral Implantology, Post Graduate Institute of Dental Sciences, Rohtak, Haryana India

## Abstract

**Introduction:**

Dryness is known to be associated with inflammatory diseases such as dry eye disease and atopic dermatitis. There is significant water loss from the oral cavity during mouth breathing. This study is conducted to estimate the influence of mouth breathing on the outcome of scaling and root planing (SRP) in chronic periodontitis (CP).

**Materials and methods:**

CP patients comprising of 33 mouth breathers (MBs) and 33 nose breathers (NBs) were recruited. Thirty patients in each group completed the study. At baseline, plaque index (PI), gingival index (GI), bleeding on probing (BOP), probing depth (PD), and clinical attachment level (CAL) were measured. SRP was done in both groups. At the 4th, 8th, and 12th week, PI, GI, and BOP were recorded. PD and CAL were also assessed at the 12th week.

**Results:**

At the 12th week, there was significantly less improvement in GI at palatal sites of maxillary anterior and maxillary posterior teeth in MB group. Sixty-nine percent of BOP positive sites with PD >4 mm were converted into BOP negative sites with PD ≤4 mm in maxillary posterior palatal sites in NB. This success was 38% in MB.

**Conclusion:**

Control of periodontal inflammation by SRP in CP patients is affected at palatal sites of mouth breathers.

## INTRODUCTION

The nose performs a major role in warming and humidifying inspired air so that inspired air is at body temperature and saturated with water vapors by the time it reaches the alveoli. As expired air passes through the nose, water is again replenished to the nasal mucosa. However, some water loss occurs in expired air. In mouth breathers (MBs), the amount of water lost in expired air is 42% more than in nose breathers (NBs).^1^

Dryness is linked to various inflammatory conditions such as dry eye disease, exercise-induced asthma, and atopic dermatitis. In dry eye disease, decreased formation of tears or increased rate of evaporation causes inflammation of the ocular surface and associated damage.^2^ During exercise, lower airways are recruited in conditioning inspired air, especially at times of increased ventilation and prolonged duration of exercise.^3^ This process of humidifying typically cold and dry air results in dehydration.^3^ Dehydration evokes release of inflammatory mediators and thereby leads to exercise-induced bronchoconstriction and asthma.^4,5^ Hydration status of the skin during wound healing influences expression of inflammatory signals in the epidermis with exaggerated response of pro-inflammatory cytokines in healing wounds under dry conditions compared to wounds that restore to normal healthy state in an optimally hydrated environment.^6^ Application of emollients on dry skin in individuals with or at an increased risk for developing atopic dermatitis decreases transepidermal water loss as well as improving hydration status of skin^7,8^ and providing relief in pruritis.^8^

In the oral cavity, tissues are protected from dessication by salivary mucins that bind with water and form a coating over the oral mucosa, thereby maintaining the tissue’s hydration.^9^ In individuals with xerostomia, increased plaque and gingival inflammation is reported.^10^ Mouth breathing has also been reported to play a role in gingival inflammation.^11–14^

Hydration status has been found to affect wound healing in animal studies. Fast healing response is observed in skin wounds under moist conditions.^15^ Reduced scar formation with rapid healing is noticed in palatal wounds as compared to cutaneous wounds.^16^ Licking of cutaneous wounds with saliva from submandibular and sublingual glands has been found to facilitate healing.^17^ Delayed wound healing of palatal wounds^18,19^ and gingivectomy wounds^20^ has been observed on interruption of salivary flow from major salivary glands. Various peptides contained in saliva contribute towards wound healing by restricting inflammation.^21,22^ Facilitation of nutrients supply through saliva^21^ may further aid in better and faster healing of oral wounds. Mouth breathing-associated oral dryness^23^ may be linked to evaporation of saliva.^24,25^ Adjunctive use of salivary substitutes in MBs resulted in significant improvement in periodontal inflammation in anterior regions of dentition after SRP.^26^ Mouth breathing-associated alterations in ecology of dento-gingival area and hydration status of investing tissues of the periodontium may alter healing response of periodontium after periodontal therapy. However, there is little research in the area.

Taking into consideration these observations, this study is aimed at investigating the effect of mouth breathing on the outcome of scaling and root planing (SRP) in patients with chronic periodontitis (CP).

## MATERIALS AND METHODS

### Study design, ethical aspects

This non-randomized interventional study registered at Clinical Trials (NCT03169322) was conducted in the Department of Periodontics and Oral Implantology, Post Graduate Institute of Dental Sciences (PGIDS), Rohtak, Haryana, India. The study was conducted in accordance with the ethical standards outlined in the Declaration of Helsinki 1975, as revised in 2013. The study protocol was reviewed and approved by the Ethical Committee, PGIDS, Rohtak, Haryana, India (PGIDS/IEC/2016/54). Written informed consent was obtained from each patient after explaining the nature and purpose of study.

### Study population

Of 208 patients screened from the outpatient department of Periodontics and Oral Implantology, the study was conducted in 66 CP patients (38 females and 28 males, aged 20 to 35 years; mean age: 31.30 ± 2.40 years) comprising of MBs (22 females and 11 males, mean age 31.21 ± 2.42 years) and NBs (16 females and 17 males, mean age 31.39 ± 2.41 years). Diagnosis of mouth breathing was made on the basis of patient’s awareness about his/her mouth breathing habit along with history of dry mouth in the morning on awakening. Study period was from October 2016 to September 2017.

Sample size calculation was carried out using a software (G-Power 3.0.10; Heinrich-Heine University, Dusseldorf, Dusseldorf, Germany). Assuming effect size of 0.8, statistical power of 80%, *α* error of 0.05, and allocation ratio of 1:1, the sample size of the study was calculated to be 27. To compensate for potential drop-outs, 33 patients were enrolled in each group.

#### Inclusion criteria

Systemically healthy CP individuals possessing ≥20 natural teeth and BOP prevalence >25%. CP criteria included ≥2 interproximal sites with AL ≥4 mm (not on same tooth), or ≥2 interproximal sites with PD ≥5 mm (not on same tooth).^27^

#### Exclusion criteria

1. Patients on anti-inflammatory drugs or antibiotics or history of treatment with medications known to influence periodontal status or healing such as statins, glucocorticoids, phenytoin, calcium channel blockers, immunosuppressants, bisphosphonates, or any other host modulatory drug within 6 months of commencement of study.
2. History of periodontal treatment within 1 year of inclusion in the study.
3. Current or former smokers or use of tobacco in any form.
4. Non-plaque-induced gingival lesions.
5. Patients taking drugs reported to cause xerostomia such as anti-cholinergics, anti-hypertensives, analgesics, sedatives, tranquilizing agents, and antihistamines.
6. A recent history of acute or chronic infection.
7. Patients with history of xerostomia.
8. Pregnant and lactating women and those taking oral contraceptive drugs.

### Treatment

SRP using manual scalers (Hu-Friedy) and curets (Gracey Curets, Hu-Friedy) and ultrasonic scaler (EMS, Nyon, Switzerland) was carried out by a single investigator (M.K.) in participants belonging to both study groups. Oral hygiene instructions (OHIs) were given. Patients were re-evaluated at the 4th, 8th, and 12th week after SRP. At each recall visit, plaque control was done and OHIs were reinforced.

### Clinical parameters

Plaque index (PI),^28^ gingival index (GI),^29^ bleeding on probing (BOP),^30^ probing depth (PD), and clinical attachment level (CAL) were measured using periodontal probe (PCP-UNC 15 periodontal probe Hu-Friedy, Chicago, IL, USA) at baseline. At the 4th and 8th week after SRP; PI, GI, and BOP were recorded. All periodontal parameters were again measured at the 12th week. PI and GI were recorded at four sites per tooth and BOP, PD, and CAL were recorded at six sites per tooth in all teeth except third molars. BOP was assessed in a dichotomous manner and was calculated as the percentage of sites positive for BOP. Maxillary labial gingival coverage by upper lip in resting position was also assessed in the study population.

All clinical periodontal examinations were carried out by a single, calibrated investigator (M.K.) to preclude inter-examiner variability. A calibration exercise was performed until reproducibility in >85% of measurements done on two occasions 48 h apart was achieved.

### Statistical analysis

Statistical analysis was performed using the software (SPSS; v.20, IBM, Chicago, IL, USA). Descriptive statistics in terms of mean and standard deviation (SD) were calculated for continuous variables such as age, PI, GI, BOP, PD, and CAL. Normality of the data was assessed using the Shapiro–Wilk test. Data for PI, BOP, and PD followed normal distribution, hence parametric tests were applied for these variables (paired *t* test for intragroup comparison and unpaired *t* test for intergroup comparison). Comparison of other variables, GI and CAL was made using non-parameteric tests (Wilcoxon's signed-rank test for intragroup comparison and Mann–Whitney test for intergroup comparison). Intergroup comparison of categorical variable, for example, gender was done using *χ*^2^ test. *P* < 0.05 was assumed to be level of significance.

## Results

### At baseline

Of 66 patients enrolled, 60 patients (30 MBs and 30 NBs) completed the study. All patients included in the study had normal upper lip coverage. Demographic characteristics and periodontal parameters of patients exhibited no significant difference except GI at lingual sites of mandibular anterior teeth and PD and CAL at palatal sites of maxillary anterior teeth (Table 1).

**Table 1:** Demographic variables and periodontal parameters (mean ± SD) in MB and NB groups at baseline

			MB	NB
*Demographic variables*				
Age (years)			30.93 ± 2.35	31.07 ± 2.27
Gender (F:M)			21:9	14:16
*Periodontal parameters*				
Region	Sites	Parameter	MB	NB
Whole dentition		PI	1.67 ± 0.33	1.75 ± 0.36
		GI	1.52 ± 0.33	1.50 ± 0.22
		BOP (%)	56.13 ± 13.26	55.21 ± 14.84
		PD (mm)	2.96 ± 0.38	3.11 ± 0.36
		CAL (mm)	3.02 ± 0.41	3.19 ± 0.43
Maxillary anterior teeth	Labial	PI	1.63 ± 0.49	1.47 ± 0.50
		GI	1.45 ± 0.39	1.39 ± 0.27
		BOP (%)	54.85 ± 28.71	50.19 ± 24.04
		PD (mm)	2.91 ± 0.70	3.01 ± 0.59
		CAL (mm)	2.90 ± 0.64	2.98 ± 0.55
	Palatal	PI	1.76 ± 0.45	1.65 ± 0.42
		GI	1.59 ± 0.37	1.61 ± 0.39
		BOP (%)	46.48 ± 22.29	50.19 ± 25.83
		PD (mm)	2.62 ± 0.40	2.87 ± 0.46*
		CAL (mm)	2.61 ± 0.43	2.88 ± 0.44*
Mandibular anterior teeth	Labial	PI	1.90 ± 0.59	1.94 ± 0.51
		GI	1.56 ± 0.53	1.39 ± 0.29
		BOP (%)	65.74 ± 23.89	57.22 ± 22.42
		PD (mm)	2.99 ± 0.58	3.09 ± 0.52
		CAL (mm)	3.03 ± 0.62	3.18 ± 0.61
	Lingual	PI	2.26 ± 0.68	2.19 ± 0.52
		GI	1.90 ± 0.57	1.70 ± 0.35*
		BOP (%)	57.96 ± 25.47	56.85 ± 25.05
		PD (mm)	2.39 ± 0.39	2.53 ± 0.49
		CAL (mm)	2.76 ± 0.61	2.93 ± 0.86
Maxillary posterior teeth	Buccal	PI	1.52 ± 0.44	1.76 ± 0.52
		GI	1.44 ± 0.34	1.55 ± 0.34
		BOP (%)	62.41 ± 22.83	66.34 ± 21.19
		PD (mm)	3.30 ± 0.52	3.46 ± 0.66
		CAL (mm)	3.33 ± 0.51	3.46 ± 0.61
	Palatal	PI	1.85 ± 0.32	1.77 ± 0.30
		GI	1.68 ± 0.32	1.70 ± 0.33
		BOP (%)	50.52 ± 20.46	56.47 ± 19.68
		PD (mm)	3.04 ± 0.55	3.26 ± 0.64
		CAL (mm)	3.01 ± 0.59	3.27 ± 0.63
Mandibular posterior teeth	Buccal	PI	1.37 ± 0.35	1.57 ± 0.44
		GI	1.33 ± 0.37	1.38 ± 0.22
		BOP (%)	51.05 ± 25.80	45.53 ± 18.38
		PD (mm)	2.91 ± 0.55	3.09 ± 0.47
		CAL (mm)	2.99 ± 0.57	3.13 ± 0.48
	Lingual	PI	2.20 ± 0.49	2.20 ± 0.48
		GI	1.81 ± 0.24	1.74 ± 0.34
		BOP (%)	59.54 ± 18.32	57.26 ± 21.46
		PD (mm)	3.32 ± 0.56	3.36 ± 0.37
		CAL (mm)	3.37 ± 0.60	3.51 ± 0.54

^*^P < 0.05

### Improvement in PI

At the 4th, 8th, and 12th week PI values in different regions and in whole dentition (WD) were significantly lower than baseline values in both groups (Tables 2 and 3). Intergroup comparison at the 4th week revealed significantly lower improvement in PI in WD and at labial sites of maxillary and mandibular posterior teeth in the MB group (Table 4). At other regions, improvement of PI between two groups revealed no significant difference (Table 4). At the 8th week and 12th week, there was no significant difference in improvement of PI between two groups (Table 4).

**Table 2:** Comparative evaluation of periodontal parameters (mean ± SD) in different regions of mouth at 4th week, 8th week, and 12th week with reference to parameters at baseline in the MB group

	Sites	Parameter	Baseline	4th week	8th week	12th week
Whole dentition		PI	1.67 ± 0.33	1.18 ± 0.31*	0.99 ± 0.37*	0.90 ± 0.38*
		GI	1.52 ± 0.33	1.23 ± 0.21*	1.05 ± 0.23*	0.94 ± 0.27*
		BOP (%)	56.13 ± 13.26	39.44 ± 16.66*	28.38 ± 10.75*	24.45 ± 9.65*
		PD (mm)	2.96 ± 0.38			2.39 ± 0.49*
		CAL (mm)	3.02 ± 0.41			2.50 ± 0.55*
Maxillary anterior teeth	Labial	PI	1.63 ± 0.49	1.09 ± 0.37*	0.93 ± 0.52*	0.87 ± 0.47*
		GI	1.45 ± 0.39	1.21 ± 0.22*	1.02 ± 0.33*	0.90 ± 0.33*
		BOP (%)	54.85 ± 28.71	32.11 ± 18.26*	27.85 ± 15.39*	24.44 ± 11.63*
		PD (mm)	2.91 ± 0.70			2.39 ± 0.54*
		CAL (mm)	2.90 ± 0.64			2.43 ± 0.55*
	Palatal	PI	1.76 ± 0.45	1.21 ± 0.42*	1.13 ± 0.35*	0.97 ± 0.44*
		GI	1.59 ± 0.37	1.33 ± 0.40*	1.23 ± 0.45*	1.12 ± .32*
		BOP (%)	46.48 ± 22.29	35.74 ± 20.51*	25.33 ± 16.22*	20.55 ± 14.60*
		PD (mm)	2.62 ± 0.40			2.21 ± 0.52*
		CAL (mm)	2.61 ± 0.43			2.24 ± 0.56*
Mandibular anterior teeth	Labial	PI	1.90 ± 0.59	1.22 ± 0.54*	1.03 ± 0.48*	0.95 ± 0.50*
		GI	1.56 ± 0.53	1.20 ± 0.29*	1.01 ± 0.28*	0.89 ± 0.29*
		BOP (%)	65.74 ± 23.89	37.96 ± 22.61*	30.37 ± 18.26*	22.04 ± 13.80*
		PD (mm)	2.99 ± 0.58			2.21 ± 0.56*
		CAL (mm)	3.03 ± 0.62			2.44 ± 0.73*
	Lingual	PI	2.26 ± 0.68	1.25 ± 0.46*	1.27 ± 0.46*	1.08 ± 0.49*
		GI	1.90 ± 0.57	1.37 ± 0.50*	1.21 ± 0.40*	1.09 ± 0.42*
		BOP (%)	57.96 ± 25.47	41.11 ± 24.39*	21.30 ± 16.77*	19.45 ± 14.28*
		PD (mm)	2.39 ± 0.39			1.89 ± 0.48*
		CAL (mm)	2.76 ± 0.61			2.31 ± 0.72*
Maxillary posterior teeth	Buccal	PI	1.52 ± 0.44	1.12 ± 0.35*	0.88 ± 0.40*	0.80 ± 0.41*
		GI	1.44 ± 0.34	1.20 ± 0.28*	0.99 ± 0.29*	0.86 ± 0.38*
		BOP (%)	62.41 ± 22.83	45.94 ± 19.77*	30.72 ± 13.15*	27.33 ± 12.66*
		PD (mm)	3.30 ± 0.52			2.77 ± 0.53*
		CAL (mm)	3.33 ± 0.51			2.82 ± 0.55*
	Palatal	PI	1.85 ± 0.32	1.51 ± 0.41*	1.20 ± 0.46*	1.08 ± 0.41*
		GI	1.68 ± 0.32	1.49 ± 0.33*	1.36 ± 0.29*	1.23 ± 0.35*
		BOP (%)	50.52 ± 20.46	42.65 ± 23.81	31.85 ± 15.09*	25.44 ± 16.88*
		PD (mm)	3.04 ± 0.55			2.51 ± 0.67*
		CAL (mm)	3.01 ± 0.59			2.56 ± 0.70*
Mandibular posterior teeth	Buccal	PI	1.37 ± 0.35	1.03 ± 0.40*	0.84 ± 0.46*	0.77 ± 0.48*
		GI	1.33 ± 0.37	1.07 ± 0.33*	0.88 ± 0.37*	0.79 ± 0.39*
		BOP (%)	51.05 ± 25.80	29.48 ± 16.55*	24.34 ± 15.96*	20.50 ± 13.65*
		PD (mm)	2.91 ± 0.55			2.28 ± 0.54*
		CAL (mm)	2.99 ± 0.57			2.38 ± 0.61*
	Lingual	PI	2.20 ± 0.49	1.56 ± 0.47*	1.32 ± 0.36*	1.19 ± 0.48*
		GI	1.81 ± 0.24	1.45 ± 0.39*	1.31 ± 0.34*	1.24 ± 0.41*
		BOP (%)	59.54 ± 18.32	47.53 ± 24.57	33.14 ± 16.76*	32.71 ± 15.25*
		PD (mm)	3.32 ± 0.56			2.65 ± 0.66*
		CAL (mm)	3.37 ± 0.60			2.72 ± 0.72*

^*^P < 0.05

**Table 3:** Comparative evaluation of periodontal parameters (mean ± SD) in different regions of mouth at 4th week, 8th week and 12th week with reference to parameters at baseline in the NB group

	Parameter	Baseline	4th week	8th week	12th week
Whole dentition	PI	1.75 ± 0.36	1.01 ± 0.36*	0.97 ± 0.35*	0.85 ± 0.38*
	GI	1.50 ± 0.22	1.10 ± 0.25*	1.03 ± 0.25*	0.84 ± 0.35*
	BOP (%)	55.21 ± 14.84	29.33 ± 11.38*	27.20 ± 11.38*	21.48 ± 13.66*
	PD (mm)	3.11 ± 0.36			2.50 ± 0.58*
	CAL (mm)	3.19 ± 0.43			2.63 ± 0.68*
*Maxillary anterior teeth*					
Labial	PI	1.47 ± 0.50	0.85 ± 0.44*	0.89 ± 0.44*	0.74 ± 0.45*
	GI	1.39 ± 0.27	0.99 ± 0.30*	0.87 ± 0.43*	0.74 ± 0.42*
	BOP (%)	50.19 ± 24.04	20.00 ± 14.63*	19.07 ± 15.96*	16.11 ± 17.28*
	PD (mm)	3.01 ± 0.59			2.40 ± 0.72*
	CAL (mm)	2.98 ± 0.55			2.42 ± 0.72*
Palatal	PI	1.65 ± 0.42	0.96 ± 0.45*	0.97 ± 0.44*	0.82 ± 0.44*
	GI	1.61 ± 0.39	1.23 ± 0.45*	1.06 ± 0.45*	0.77 ± 052*
	BOP (%)	50.19 ± 25.83	26.67 ± 15.60*	24.26 ± 18.02*	16.67 ± 16.18*
	PD (mm)	2.87 ± 0.46			2.18 ± 0.58*
	CAL (mm)	2.88 ± 0.44			2.21 ± 0.58*
*Mandibular anterior teeth*					
Labial	PI	1.94 ± 0.51	1.09 ± 0.37*	1.02 ± 0.39*	0.90 ± 0.39*
	GI	1.39 ± 0.29	1.07 ± 0.24*	1.06 ± 0.33*	0.87 ± 0.40*
	BOP (%)	57.22 ± 22.42	31.30 ± 20.55*	26.11 ± 19.31*	19.81 ± 14.34*
	PD (mm)	3.09 ± 0.52			2.39 ± 0.64*
	CAL (mm)	3.18 ± 0.61			2.59 ± 0.81*
Lingual	PI	2.19 ± 0.52	1.14 ± 0.50*	1.22 ± 0.44*	0.98 ± 0.46*
	GI	1.70 ± 0.35	1.09 ± 0.42*	1.08 ± 0.51*	0.80 ± 0.51*
	BOP (%)	56.85 ± 25.05	25.74 ± 18.77*	19.82 ± 13.89*	13.70 ± 13.82*
	PD (mm)	2.53 ± 0.49			2.06 ± 0.62*
	CAL (mm)	2.93 ± 0.86			2.53 ± 0.93*
*Maxillary posterior teeth*					
Buccal	PI	1.76 ± 0.52	0.97 ± 0.46*	0.97 ± 0.38*	0.85 ± 0.42*
	GI	1.55 ± 0.34	1.10 ± 0.33*	1.04 ± 0.32*	0.85 ± 0.36*
	BOP (%)	66.34 ± 21.19	32.14 ± 12.95*	31.22 ± 16.16*	24.60 ± 15.70*
	PD (mm)	3.46 ± 0.66			2.86 ± 0.73*
	CAL (mm)	3.46 ± 0.61			2.91 ± 0.76*
Palatal	PI	1.77 ± 0.30	1.24 ± 0.52*	1.13 ± 0.53*	0.94 ± 0.49*
	GI	1.70 ± 0.33	1.35 ± 0.41*	1.30 ± 0.45*	0.97 ± 0.49*
	BOP (%)	56.47 ± 19.68	36.40 ± 21.02*	33.27 ± 18.46*	26.87 ± 20.06*
	PD (mm)	3.26 ± 0.64			2.61 ± 0.81*
	CAL (mm)	3.27 ± 0.63			2.66 ± 0.83*
*Mandibular posterior teeth*					
Buccal	PI	1.57 ± 0.44	0.93 ± 0.40*	0.78 ± 0.47*	0.77 ± 0.47*
	GI	1.38 ± 0.22	1.06 ± 0.27*	0.95 ± 0.33*	0.82 ± 0.44*
	BOP (%)	45.53 ± 18.38	25.79 ± 14.93*	24.88 ± 15.07*	22.82 ± 19.61*
	PD (mm)	3.09 ± 0.47			2.57 ± 0.69*
	CAL (mm)	3.13 ± 0.48			2.68 ± 0.80*
Lingual	PI	2.20 ± 0.48	1.32 ± 0.56*	1.25 ± 0.47*	1.01 ± 0.48*
	GI	1.74 ± 0.34	1.21 ± 0.51*	1.21 ± 0.42*	1.04 ± 0.38*
	BOP (%)	57.26 ± 21.46	33.49 ± 18.82*	32.94 ± 16.37*	27.20 ± 15.71*
	PD (mm)	3.36 ± 0.37			2.71 ± 0.67*
	CAL (mm)	3.51 ± 0.54			2.86 ± 0.79*

^*^P < 0.05

**Table 4:** Comparison of improvement (∆) in periodontal parameters (mean ± SD) at 4th week, 8th week, and 12th week between two groups

Region	Parameter	0–4th week		0–8th week		0–12th week	
		MB group	NB group	MB group	NB group	MB group	NB group
Whole dentition							
	∆PI	0.49 ± 0.39	0.74 ± 0.44*	0.68 ± 0.47	0.78 ± 0.47	0.77 ± 0.44	0.90 ± 0.51
	∆GI	0.29 ± 0.28	0.40 ± 0.25	0.47 ± 0.31	0.48 ± 0.24	0.58 ± 0.35	0.66 ± 0.30
	∆BOP (%)	16.69 ± 15.84	25.88 ± 17.71*	27.75 ± 15.53	28.01 ± 17.15	31.68 ± 15.36	33.72 ± 17.18
	∆PD (mm)					0.57 ± 0.39	0.61 ± 0.47
	∆CAL (mm)					0.52 ± 0.38	0.56 ± 0.48
*Maxillary anterior teeth*							
Labial	∆PI	0.54 ± 0.46	0.62 ± 0.50	0.71 ± 0.68	0.58 ± 0.44	0.77 ± 0.62	0.72 ± 0.50
	∆GI	0.24 ± 0.34	0.40 ± 0.30*	0.43 ± 0.48	0.51 ± 0.38	0.55 ± 0.47	0.64 ± 0.38
	∆BOP (%)	22.74 ± 30.55	30.18 ± 26.93	27.00 ± 32.46	31.11 ± 27.32	30.41 ± 27.64	34.07 ± 26.81
	∆PD (mm)					0.53 ± 0.51	0.60 ± 0.44
	∆CAL (mm)					0.47 ± 0.47	0.56 ± 0.43
Palatal	∆PI	0.55 ± 0.51	0.69 ± 0.47	0.63 ± 0.51	0.68 ± 0.59	0.80 ± 0.59	0.83 ± 0.56
	∆GI	0.26 ± 0.46	0.38 ± 0.55	0.36 ± 0.43	0.54 ± 0.58	0.47 ± 0.46	0.84 ± 0.54*
	∆BOP (%)	10.74 ± 27.56	23.52 ± 28.31	21.15 ± 20.47	25.93 ± 30.16	25.93 ± 19.15	33.52 ± 25.50
	∆PD (mm)					0.41 ± 0.42	0.68 ± 0.57*
	∆CAL (mm)					0.38 ± 0.40	0.67 ± 0.56
*Mandibular anterior teeth*							
Labial	∆PI	0.68 ± 0.54	0.85 ± 0.56	0.86 ± 0.65	0.93 ± 0.60	0.95 ± 0.63	1.05 ± 0.62
	∆GI	0.36 ± 0.54	0.32 ± 0.26	0.55 ± 0.51	0.34 ± 0.37	0.67 ± 0.54	0.52 ± 0.39
	∆BOP (%)	27.78 ± 20.94	25.92 ± 25.83	35.37 ± 28.08	31.11 ± 26.33	43.70 ± 27.98	37.41 ± 25.43
	∆PD (mm)					0.78 ± 0.65	0.69 ± 0.63
	∆CAL (mm)					0.58 ± 0.58	0.60 ± 0.64
Lingual	∆PI	1.01 ± 0.69	1.05 ± 0.60	0.98 ± 0.79	0.98 ± 0.50	1.17 ± 0.82	1.22 ± 0.53
	∆GI	0.53 ± 0.56	0.61 ± 0.50	0.69 ± 0.59	0.62 ± 0.62	0.81 ± 0.57	0.90 ± 0.48
	∆BOP (%)	16.85 ± 37.24	31.11 ± 28.28	36.67 ± 25.92	37.04 ± 30.30	38.52 ± 24.19	43.15 ± 29.71
	∆PD (mm)					0.50 ± 0.46	0.47 ± 0.39
	∆CAL (mm)					0.45 ± 0.46	0.39 ± 0.42
*Maxillary posterior teeth*							
Buccal	∆PI	0.40 ± 0.47	0.79 ± 0.54*	0.64 ± 0.47	0.80 ± 0.57	0.71 ± 0.45	0.91 ± 0.61
	∆GI	0.25 ± 0.38	0.44 ± 0.30*	0.45 ± 0.40	0.50 ± 0.33	0.59 ± 0.43	0.69 ± 0.31
	∆BOP (%)	16.47 ± 21.68	34.20 ± 20.55*	31.69 ± 25.73	35.12 ± 22.75	35.08 ± 23.93	41.75 ± 20.47
	∆PD (mm)					0.53 ± 0.34	0.60 ± 0.45
	∆CAL (mm)					0.51 ± 0.32	0.55 ± 0.47
Palatal	∆PI	0.34 ± 0.40	0.53 ± 0.55	0.65 ± 0.52	0.64 ± 0.58	0.78 ± 0.48	0.82 ± 0.57
	∆GI	0.19 ± 0.39	0.36 ± 0.44*	0.32 ± 0.34	0.40 ± 0.49	0.45 ± 0.41	0.73 ± 0.36*
	∆BOP (%)	7.87 ± 28.89	20.07 ± 23.00	18.67 ± 18.14	23.20 ± 20.38	25.08 ± 20.54	29.60 ± 22.51
	∆PD (mm)					0.53 ± 0.47	0.65 ± 0.62
	∆CAL (mm)					0.46 ± 0.53	0.61 ± 0.61
*Mandibular posterior teeth*							
Buccal	∆PI	0.34 ± 0.45	0.64 ± 0.51*	0.53 ± 0.48	0.79 ± 0.66	0.60 ± 0.52	0.80 ± 0.67
	∆GI	0.27 ± 0.32	0.32 ± 0.29	0.45 ± 0.44	0.43 ± 0.29	0.54 ± 0.46	0.56 ± 0.38
	∆BOP (%)	21.57 ± 28.24	19.74 ± 22.52	26.71 ± 25.49	20.65 ± 23.80	30.55 ± 27.95	22.72 ± 26.81
	∆PD (mm)					0.64 ± 0.50	0.52 ± 0.56
	∆CAL (mm)					0.60 ± 0.47	0.45 ± 0.67
Lingual	∆PI	0.63 ± 0.63	0.88 ± 0.65	0.87 ± 0.56	0.95 ± 0.65	1.00 ± 0.68	1.19 ± 0.68
	∆GI	0.36 ± 0.39	0.53 ± 0.51	0.50 ± 0.42	0.53 ± 0.49	0.57 ± 0.47	0.70 ± 0.44
	∆BOP (%)	12.02 ± 32.59	23.77 ± 29.01	26.40 ± 22.92	24.33 ± 29.48	26.84 ± 21.11	30.06 ± 26.73
	∆PD (mm)					0.67 ± 0.62	0.65 ± 0.71
	∆CAL (mm)					0.65 ± 0.61	0.64 ± 0.71

^*^P < 0.05

### Improvement in GI

In both groups, GI decreased significantly at the 4th, 8th, and 12th week as compared to baseline GI scores in different regions and in WD (Tables 2 and 3). At the 4th week, significantly lower improvement in GI was found at labial sites of maxillary anterior teeth, buccal, and palatal sites of maxillary posterior teeth in the MB group (Table 4). Intergroup comparison of GI in other regions exhibited no significant difference (Table 4). At the 8th week, improvement in GI was comparable between the two groups in different regions of dentition (Table 4). At the 12th week, significantly lesser improvement was seen in GI scores at palatal sites of maxillary anterior and maxillary posterior teeth in the MB group (Table 4).

### Improvement in BOP

Sites of various regions exhibited significant reduction in BOP at all recall visits with respect to baseline BOP in both groups except palatal and lingual sites of posterior teeth at the 4th week in the MB group (Tables 2 and 3). At 4th week, BOP scores in WD and BOP at buccal sites of maxillary posterior teeth had significantly lesser improvement in the MB group as compared to the NB group (Table 4). Other regions exhibited no significant difference (Table 4). At the 8th week and 12th week, no significant difference between two groups was found in improvement of BOP (Table 4).

### Improvement in PD and CAL

In both groups, PD and CAL reduced significantly at the 12th week as compared to their baseline values (Tables 2 and 3). Intergroup comparison of improvement in PD and CAL in WD and in different regions of dentition revealed no significant differences (Table 4), with the exception of higher improvement in PD at palatal sites of maxillary anterior teeth in the NB group.

## DISCUSSION

Apart from providing a moist environment, saliva contains various components that play a key role in wound healing. Qualitative or quantitative changes in saliva influence healing of oral mucosal wounds. Furthermore, ecological changes during mouth breathing may influence healing of soft tissues bordering the teeth. This is the first prospective study conducted to explore the effect of mouth breathing on the outcome of SRP in CP.

In the present study, at baseline, patients belonging to both groups had comparable PI, GI, and BOP. Stringent inclusion and exclusion criteria including BOP score >25% may be partly responsible for this finding.

In both groups, significant reduction in WD PI, GI, and BOP was found at all recall visits in comparison to baseline values. Continuous improvement in PI at each recall visit until the 12th week may be attributed to professional plaque control and reinforcement of OHIs at each recall visit. Accordingly, GI and BOP also continued to reduce until the 12th week after SRP.

As expected, WD PD, and CAL revealed significant reduction at 12 weeks after SRP in both groups. This improvement is generally attributed to resistance offered to penetration of probe owing to reduction in infiltrated connective tissues^31,32^ along with new collagen formation in periodontal tissues.^31^

In the present study, at the 4th week after SRP, improvement in full mouth PI and BOP were significantly lower in MB as compared to NB. Oral dryness coupled with lowering of local defense served by salivary constituents may be responsible for less improvement in plaque control and periodontal inflammation in MB. Since BOP represents inflammation of underlying periodontal tissues, significantly lesser improvement of BOP at the 4th week may hint towards the possibility of short-term affect of mouth breathing on resolution of inflammation and thus healing of periodontium. Non-significant difference in improvement of GI between groups (0.29 versus 0.40) does not rule out impact of mouth breathing on control of inflammation of marginal gingiva through SRP as difference does not seems to be clinically irrelevant.

Intergroup comparison at the 4th week revealed that labial sites of maxillary anterior region in MB had significantly lesser improvement in GI. In this study, although all MB had complete upper lip coverage, frictional activity of upper lip may be reduced in MB as salivary coating on labial mucosa is found to be minimal,^33^ which may further be affected by mouth breathing-associated water loss from the oral cavity.

In the mandibular anterior region, improvement in PI, GI, and BOP was almost comparable. Better lower lip coverage of mandibular anterior teeth coupled with more availability of saliva in this region may be responsible for dampening the effect of mouth breathing on salivary coating of investing tissues, thereby resulting in similar outcomes in this region.

Intergroup comparison at 4th week revealed that MB maxillary posterior labial sites had significantly less improvement in PI, GI, and BOP, whereas palatal sites exhibited significant difference in GI improvement without any significant difference in improvement of PI. This finding suggests the possibility of some factor other than amount of plaque which may also be influencing the control of inflammation after SRP. Surface moisture level of the hard palate is affected more severely during mouth breathing as compared to other parts of the oral cavity^25^ as it is found to be the least wet oral mucosal site.^33^ Other possible explanation for this finding may be that hard palate encounters most of the air during mouth breathing.^24^ The amount of water loss during evaporation may reach upto 0.21 mL/min.^24^

Intergroup comparison revealed no significant difference in improvement of PI, GI, and BOP in WD as well as in different regions of dentition at the 8th week. At the 12th week, improvement in WD PI, GI, BOP, PD, and CAL was similar in both groups. This represents that control of periodontal inflammation may be slow in the initial period in MB, but it is not affected if similar plaque control is achieved after SRP. Comparable improvement in inflammatory parameters in the two groups, suggestive of similar degree of healing, may be responsible for identical improvement in terms of PD and CAL in WD.

During extraction. wound healing in sialadenectomized rats, delayed bone formation,^34–36^ slower replacement of clot by granulation tissue, pronounced inflammation, and initial slower rate of formation of fibrous connective tissue has been observed.^34,35^ Healing of excisional palatal wounds in desalivated rats is reported to have more intense inflammation together with limited new connective tissue formation,^18,19^ with larger size palatal wounds affected more than smaller palatal wounds.^19^ Another study in sialadenectomized rats found delayed healing of gingivectomy wound.^20^ Healing may be retarded because of dryness and lack of growth factors and other peptides that aid in wound healing.

At the 12th week, improvement in GI of maxillary anterior palatal as well as maxillary posterior palatal sites was significantly less in MB without any significant difference in improvement in PI. This finding suggests that the putative disturbance in the hydration homeostasis of palatal sites during mouth breathing may either be directly responsible for some component of gingival inflammation or may act as a modifying factor responsible for inflammation in the presence of plaque. Intergroup comparison also revealed significant difference in improvement of PDs of palatal sites in maxillary anterior region. As deep pockets undergo more improvement than shallow pockets after SRP,^37^ the presence of significantly deeper pockets in this region among NB at baseline may be a reason for better improvement in NB. Furthermore, reduced improvement in GI could also have contributed partly to this finding.

Sites with high PD at maintenance visits have increased risk for further disease progression.^38–40^ High negative predictive value for further clinical attachment loss is also associated with the absence of BOP.^30^ However these parameters when considered together are likely to be more reliable clinical endpoints of treatment success in SRP.^41^ Hence sites with the absence of BOP and PD ≤4 mm can be considered to indicate treatment success in non-surgical periodontal therapy.^41^ At the 12th week after SRP, 69% maxillary posterior palatal sites (38 out of 55 sites) with PD >4 mm and the presence of BOP were converted into sites with PD ≤4 mm without BOP in the NB group as compared to 38% (14 out of 37 sites) improvement in a similar fashion in the MB group. In maxillary anterior palatal sites, these results were 65% (11 out of 17 sites) and 57% (4 out of 7 sites) in the NB group and MB group, respectively.

Strengths of the study include strict inclusion and exclusion criteria, thereby minimizing the confounders that might affect the results of this study. Patients included in both groups belonged to the same ethnic background and have comparable demographic variables at baseline. There are some limitations of the study that include lack of assessment of microbial analysis of plaque and hydration status of investing tissues. Local inflammatory markers were also not assessed in this study.

## CONCLUSION

Within the limits of this study, it is concluded that control of periodontal inflammation with SRP in MB may be slow initially without affecting the overall results at 12 weeks. However, the outcome of SRP may be affected in maxillary palatal sites of MB. It would be beneficial to current knowledge if long-term clinical studies are conducted with inclusion of microbial assessment of plaque and analyses of hydration state of gingival tissues.
